# Identification of Asiaticoside from *Centella erecta* (Apiaceae) as Potential Apyrase Inhibitor by UF-UHPLC-MS and Its In Vivo Antischistosomal Activity

**DOI:** 10.3390/pharmaceutics14051071

**Published:** 2022-05-17

**Authors:** Lara Soares Aleixo de Carvalho, Vinícius Carius de Souza, Vinícius C. Rodrigues, Aline Correa Ribeiro, Jorge Willian Leandro Nascimento, Priscila V. S. Z. Capriles, Priscila de F. Pinto, Josué de Moraes, Ademar Alves da Silva Filho

**Affiliations:** 1 Faculdade de Farmácia, Departamento de Ciências Farmacêuticas, Universidade Federal de Juiz de Fora, R. José Lourenço Kelmer s/n, Campus Universitário, Juiz de Fora 36036-900, MG, Brazil; larasoares86@gmail.com; 2 Programa de Pós-Graduação em Modelagem Computacional, Departamento de Ciência da Computação, Instituto de Ciências Exatas, Universidade Federal de Juiz de Fora, Juiz de Fora 36036-900, MG, Brazil; carius.nara@gmail.com (V.C.d.S.); capriles@ice.ufjf.br (P.V.S.Z.C.); 3 Núcleo de Pesquisa em Doenças Negligenciadas, Universidade Guarulhos, Guarulhos 07023-070, SP, Brazil; castrov25@outlook.com (V.C.R.); moraesnpdn@gmail.com (J.d.M.); 4 Instituto de Ciências Biológicas, Universidade Federal de Juiz de Fora, Juiz de Fora 36036-900, MG, Brazil; alinecorrearibeiro@yahoo.com.br (A.C.R.); jorge.willian@ufjf.br (J.W.L.N.); priscila.faria@ufjf.br (P.d.F.P.)

**Keywords:** *Schistosoma mansoni*, natural products, asiaticoside

## Abstract

Schistosomiasis, caused by parasites of the genus *Schistosoma*, is a neglected disease with high global prevalence, affecting more than 240 million people in several countries. Praziquantel (PZQ) is the only drug currently available for the treatment. *S. mansoni* NTPDases (known as SmNTPDases, ATP diphosphohydrolases or *ecto*-apyrases) are potential drug targets for the discovery of new antischistosomal drugs. In this study, we screen NTPDases inhibitors from *Centella erecta* (Apiaceae) using an ultrafiltration combined UHPLC-QTOF-MS method and potato apyrase, identifying asiaticoside as one of the apyrase-binding compounds. After isolation of asiaticoside from *C. erecta* extract, we assessed its in vivo antischistosomal activities against *Schistosoma mansoni* worms and its in vitro enzymatic apyrase inhibition. Also, molecular docking analysis of asiaticoside against potato apyrase, *S. mansoni* NTPDases 1 and 2 were performed. Asiaticoside showed a significant in vitro apyrase inhibition and molecular docking studies corroborate with its possible actions in potato apyrase and *S. mansoni* NTPDases. In mice harboring a patent *S. mansoni* infection, a single oral dose of asiaticoside (400 mg/kg. p.o.) showed significantly in vivo antischistosomal efficacy, markedly decreasing the total worm load and egg burden, giving support for further exploration of apyrase inhibitors as antischistosomal agents.

## 1. Introduction

Schistosomiasis, caused by helminth parasites of the genus *Schistosoma*, is a neglected tropical disease with an estimated global prevalence of 240 million infected people in 78 countries and significant mortality and social and economic consequences [1,2,3]. Praziquantel (PZQ) is the only drug currently available for the treatment of all *Schistosoma* species [2,3,4,5], including *Schistosoma mansoni*, which is the species prevalent in South America, the Middle East, and Africa [1,3,6]. However, despite its effectiveness, PZQ has no efficacy against juvenile worms, which is a limit to schistosomiasis control and elimination [1,4,5]. Additionally, the emerging PZQ-resistance and its reduced efficacy after use over the last decades have also been reported [1,2,3]. Therefore, the need for identification of new therapeutic targets and antischistosomal drugs is crucial.

In this regard, *S. mansoni* NTPDases (known as ATP diphosphohydrolases, ecto-apyrases or SmNTPDases) have gained interest as potential drug target for discovering of new antischistosomal drugs [1,2,3,7,8]. It has been suggested that SmNTPDases inhibitors might act in *Schistosoma* due to several possible mechanisms, such as reducing the parasite’s survival and improving the host’s immune response [1,2,7,8]. Additionally, previous studies have showed that the SmNTPDases isoforms have immune cross-reactivity with the potato (*Solanum tuberosum*) apyrase [9,10,11], sharing high homology and similarity in their active sites [9,10,11,12]. Regarding this, some natural compounds, such as cardamonin, have promoted the inhibition of both SmNTPDases and potato apyrase, also showing in vivo antischistosomal effects against *S. mansoni* [13,14,15]. Since its obtainment with high purity is easy, it has been proposed that potato apyrase may be used in the initial screening for selecting potential antischistosomal compounds with affinity for SmNTPDases in complex samples, such as plant extracts [13,15]. In this regard, in a preliminary assessment of some plant extracts against potato apyrase, the hydroalcoholic extract of *Centella erecta* (L.f.) Fern. (Apiaceae) inhibited apyrase activity by around 32% when tested at 100 µg/mL (data not shown). *Centella* species are used in folk medicines of all over the world for many purposes, such as for the treatment of venous insufficiency and some infectious, liver, and inflammatory diseases [16]. While *C. asiatica* is the most common species in numerous herbal products in the global market [16], *C. erecta* is a closely related species of *C. asiatica* that is found in North America and in South America, such as Brazil [16].

Additionally, in recent years, ultrafiltration coupled to liquid chromatography-mass spectrometry has been proven useful as screening assay and powerful tool to reduce time and cost for the identification of naturally occurring enzyme inhibitors [17,18,19].

In this study, we have used an ultrafiltration combined UHPLC-QTOF-MS method to screen apyrase inhibitors from *C. erecta*. After identified asiaticoside as a potential apyrase ligand, its inhibitory activity on potato apyrase and its in vivo antischistosomal properties, against *S. mansoni*, were evaluated. Additionally, molecular docking analysis of potato apyrase, SmNTPDase 1, and SmNTPDase 2 with asiaticoside were investigated.

## 2. Materials and Methods

### 2.1. Chemicals and Reagents

Cardamonin (95% purity, used as a reference apyrase ligand) was obtained as previously described [13]. Methanol and acetonitrile of HPLC grade were purchased from Merck Company Inc. (Darmstadt, Germany). Formic and phosphoric acids of HPLC grade was purchased from Aladdin Industrial Corporation (Shanghai, China). Water for LC–MS/MS was prepared by Milli-Q water purification system (Millipore, Bedford, United States). Centrifugal ultrafiltration filters (Amicon Ultra-0.5, 10 kD) were purchased from Millipore Co., Ltd. (Bedford, MA, USA). Potato apyrase was purified from commercial potato (*Solanum tuberosum*) according to method previously described [15]. PZQ was provided by Ecovet Indústria Veterinária Ltda (São Paulo, Brazil). Roswell Park Memorial Institute (RPMI 1640) culture medium and inactivated fetal bovine serum were purchased from Vitrocell (Campinas, Brazil). HEPES buffer, glutaraldehyde solution and dimethyl sulfoxide (DMSO) were obtained from Sigma-Aldrich (St. Louis, MO, USA). All other chemicals and solvents were of analytical grade.

### 2.2. Plant Extraction and Preparation

Aerial parts of *Centella erecta* (Apiaceae) were collected at Faculty of Pharmacy’s medicinal herb garden, 21°46′38.7″ S 43°22′00.5″ W, Juiz de Fora city, MG, Brazil, in January 2018. Voucher specimen of *C. erecta* (CESJ 65907) was identified and stored at the herbarium of the Botany Department of the Federal University of Juiz de Fora. This study was developed in line with Brazilian federal law number 13.123/2015 on access to genetic heritage, registered under number AE32DB3. After collection, plant material was dried at 40 °C, pulverized, and extracted, by maceration, using ethanol: H_2_O (9:1 *v*/*v*, 2 L) as solvent. Next, solvent was removed under reduced pressure to yield the crude hydroalcoholic extract of *C. erecta* (Ce).

### 2.3. Optimal Incubation Conditions with Cardamonin and Potato Apyrase by HPLC-DAD

To study the optimal incubation conditions, the influence of enzyme concentrations and incubation time were investigated. Cardamonin (400 μM), used as apyrase inhibitor [13], was incubated using three different concentrations of apyrase (6, 18 and 24 μg/mL) for 120 min, in a total volume of 400 μL, to evaluate the influence of enzyme concentration on the binding degrees. To determine the appropriate incubation time for the assays, cardamonin (400 μM) was incubated at various time points (40, 80 and 120 min). Cardamonin (400 μM) was also incubated without the apyrase enzyme for each experiment in a total 400 μL to investigated non-specific binding. After incubation, the mixture was transferred for a centrifugal ultrafiltration filter, containing a regenerated cellulose ultrafiltration membrane with 10 kDa molecule weight cut-off, and filtered by centrifugation at 13000× *g* for 20 min. After ultrafiltration assays, the obtained ultrafiltrates were analyzed by HPLC-DAD.

The obtained ultrafiltrates of cardamonin were initially dried in a water bath at 40 °C ± 5 °C and nitrogen gas, for approximately 2 h. Then, the solution was resuspended in 300 mL of HPLC grade methanol, centrifuged at 10,000 rpm, filtered using a 0.45 μm filter, and injected (30 µL). After, ultrafiltrates were analyzed using high performance liquid chromatography (HPLC) (Waters Corporation, Milford, MA, USA) equipped with DAD (diode array detection) (Waters 2998), binary HPLC pump (Waters 1525), and an autosampler (Waters 2707). A SunFire C_18_ column (5 μm particle size, 4.6 mm × 250 mm, Waters) with a SunFire C_18_ precolumn (5 μm particle size, 4.6 mm × 20 mm, Waters) was used as analytical column. The mobile phase was a mixture of ultrapure water acidified with 0.5% of phosphoric acid (A) and methanol (B). The gradient method was as follows: 0–40 min, 40–100% B; 40–45 min, 100% B; 45–46 min 100–40% B; and 46–60 min, 40% B, with the flow rate of 1 mL/min with detection at λ 345 nm.

The binding of cardamonin and apyrase was measured by the binding degree (%) [20], which can be calculated by Equation (1).

(1) $$\mathrm{Binding}\;\mathrm{degree}\;\%=\frac{Aa-Ab}{Aa}\times 100$$

where *Aa* and *Ab* are the peak areas of cardamonin interacting with and without apyrase, respectively, in the HPLC-DAD chromatograms.

### 2.4. Screening of Apyrase Inhibitors by Ultrafiltration and UHPLC-QTOF-MS-Based Binding Assay

The screening experiment was performed in an ultrafiltration UHPLC-QTOF-MS system according to methods previously described [18,20,21], with some modifications. A volume of 40 μL of *C. erecta* sample solution (40 mg/mL, MeOH: H_2_O 1:1 *v*/*v*) was incubated for 120 min, at 37 °C, with 15 μL of apyrase (24 μg/mL, dissolved in phosphate buffer, pH 6.8), in a total volume of 400 μL. After incubation, the mixture was transferred for a centrifugal ultrafiltration filter containing a regenerated cellulose ultrafiltration membrane with 10 kDa molecule weight cut-off and filtered by centrifugation at 13,000× *g* for 20 min. To remove unbound compounds, filters were washed three times by centrifugation with 200 μL aliquots of phosphate buffer (pH 6.8). The bound ligands were released by adding 800 μL of methanol followed by centrifugation. Then, ultrafiltrates were injected directly into the UHPLC-QTOF-MS system (Waters Corporation, Milford, MA, USA).

### 2.5. Annotation of Potential Apyrase Ligands by Ultrafiltration Coupled to UHPLC-ESI-QTOF-MS

The annotation of potential apyrase ligands in *C. erecta* ultrafiltrates was carried by ultraperformance liquid chromatograph (UHPLC) analysis using an Acquity UPLC system (Waters Corporation, Milford, MA, USA) equipped with a binary pump, inline degasser, and autosampler coupled to an electrospray ionization quadrupole time-of-flight tandem mass spectrometer (ESI-Q TOF/MS) (Waters Corporation, Milford, MA, USA). Separation was carried out on BEH C_18_ column (100 mm × 2.1 mm, 1.7 μm, Milford, MA, USA). The mobile phase consisted of LC grade water with 0.1% formic acid (A) and LC grade acetonitrile (B) with the following gradient profiles: 0–2 min, 5% B; 2–14 min, 5–98% B; 14–16 min, 98% B; and 16–20 min, 98–5% B. The flow rate was 0.4 mL/min. The obtained ultrafiltrates were evaporated under a water bath at 40 °C ± 5 °C with a gentle stream of nitrogen gas, for approximately 2 h. After dryness, they were reconstituted in 300 mL of HPLC grade methanol, centrifuged at 10,000 rpm, filtered using a 0.22 μm filter, and injected (injection volume of 30 µL) to LC-MS analysis. All assays were performed in duplicate.

Mass spectrometry analysis was performed with a XEVO G2S QTOF mass spectrometer (Waters Corporation, Milford, MA, USA) with ESI operating in the negative ion mode for scanning. The scanning range was set at *m*/*z* 100–1000. Capillary voltage was 3.0 kV, the low collision energy was 6 eV, and the higher collision energy was 15–30 eV. The ion source temperature was 120 °C, and the desolvation temperature was 450 °C. Nitrogen was used as the source of desolvation gas (800 L·h^−^^1^) and cone gas (50 L·h^−1^). For accurate mass measurements, data were centroided during acquisition, and 200 pg·mL^−^^1^ of leucine-enkephalin (*m*/*z* 565.2771) (Sigma-Aldrich, Steinheim, Germany), dissolved in acetonitrile/0.1% formic acid (50:50, *v*/*v*), and was infused continuously as an external reference (LockSpray™) into the ESI source with automatic mass correction enabled. Data were processed using Chromalynx™ application manager with MassLynx™ 4.1 software (Waters Corporation, Milford, MA, USA). Alongside the observed MS spectra and data obtained by QTOF-MS analysis, the main tools for compound annotation were the interpretation of the observed QTOF-MS spectra in comparison with those found in the literature and online databases (ChemSpider, MassBank, and Spectral Database for Organic Compounds).

### 2.6. Isolation of Asiaticoside from C. erecta Extract

After collection, aerial parts of *C. erecta* were dried at 40 °C and powdered in a blender. The resulting powder (580 g) was extracted, by maceration, using ethanol: H_2_O (9:1 *v*/*v*, 4 L) as solvent. After extraction, solvent was removed under reduced pressure to yield 26 g of the crude hydroalcoholic extract (Ce) of *C. erecta*. The crude Ce extract (26 g) was suspended in methanol: H_2_O (9:1 *v*/*v*) and submitted to sequential partition with *n*-hexane (Ce-H, 1.2 g), ethyl acetate (Ce-A, 5.5 g) and butanol (Ce-B, 4.2 g). Then, Ce-B (4.2 g) was chromatographed over Sephadex LH-20, using methanol 100%, furnishing 7 fractions: fraction I (8.4 mg), fraction II (304.8 mg), fraction III (138.8 mg), fraction IV (254.0 mg), fraction V (84.0 mg), fraction VI (1073.6 mg), and fraction VII (601.6 mg). An isolated compound, obtained in fraction V, was chemically identified as asiaticoside by ^1^H and ^13^C NMR analysis. ^1^H- and ^13^C- NMR spectra were recorded in CD_3_OD solutions on a Bruker 500 Advance spectrometer (500 MHz for ^1^H NMR and 125 MHz for ^13^C NMR) with chemical shifts (δ) reported in parts per million (ppm) relative to trimethylsilane (TMS) as internal standard and coupling constants (J) in Hertz (Hz).

### 2.7. Potato Apyrase Inhibitory Assay

Enzymatic apyrase activity was evaluated according to literature [15], using the modified method of Taussky and Shorr [22]. Briefly, 0.6 ng of potato apyrase was added to the reaction medium containing 50 mM succinate buffer pH 6.5 supplemented with 5 mM CaCl_2_, in the absence or presence of asiaticoside (25, 50 and 100 µM) and samples were analyzed in triplicate. After, for stabilization of the system, tubes were incubated for 10 min at 37 °C and the reaction was triggered with the addition of 3mM of ATP and the reaction was stopped by the addition of HCl 0.1 N. Then, inorganic phosphate (Pi) released was determined by the addition of color reagent (an acidic aqueous ferrous sulfate solution plus ammonium molybdate) and the optical density was measured at 660 nm. The percentage of apyrase inhibition was calculated according to literature [15].

### 2.8. In Silico Analysis and Molecular Docking of Potato Apyrase, SmNTPDases 1 and 2

#### 2.8.1. Three-Dimensional Structure of Target Proteins

The three-dimensional (3D) model of apyrase, SmNTPDase 1, and SmNTPDase 2 used in this study were previously published [13,23,24].

#### 2.8.2. Molecular Docking Simulations

All molecular docking assays were performed by the Autodock Vina program [25] 3D models of target proteins were prepared adding hydrogen atoms to pH 7.4 and assigning Gasteiger partial charges using MGLTools 1.5.6 program [26]. We defined as flexible the side chain of some amino acids in the active site (catalytic residues and stabilization residues), described as important for the enzymatic activity of apyrase, SmNTPDase 1, and SmNTPDase 2. Ligand, ions, and non-conserved waters were removed during the protein preparation for docking simulations. The grid center (with an edge of 30Å) was set at the nucleotide-binding site (X = 22.268, Y= 34.516, and Z = 21.621). After the assay, the first ten best-scoring docking poses were analyzed. Additionally, pharmacophoric profiles of ligands and catalytic sites were analyzed using the Align-it 1.0.4 (http://silicos-it.be.s3-website-eu-west-1.amazonaws.com, accessed on 25 March 2022) plugin from PyMol software [27].

#### 2.8.3. Calculated Inhibition Constant 

The calculated inhibition constant (*cKi*) values were obtained from the interaction energy of best-docked poses for each protein-ligand complex applying the Equation (2) [28]:

(2) $$cK_{i}=e^{\left(∆G*1000\right)/RT}$$

where Δ*G* is the energy obtained from the simulation, *R* is the gas constant (1.98719 cal·K^−1^·mol^−1^), and *T* is the temperature in Kelvin (298.15 K).

### 2.9. In Vivo Antischistosomal Studies

#### 2.9.1. Animals and Parasite Maintenance

The Belo Horizonte (BH) strain of *S. mansoni*, used in all assays, was maintained by the passage through mice and *Biomphalaria glabrata* snails, as definitive and intermediate hosts, respectively, at the Núcleo de Pesquisa em Doenças Negligenciadas (São Paulo, Brazil) [29]. Female Swiss mice (weighing ~20 g each, 3 weeks old) were purchased from Animais para Laboratório (Anilab, São Paulo, Brazil). Both mice and snails were kept under environmentally controlled conditions (temperature, 25 °C; humidity, 50%) and light cycles (12-h light and 12-h dark), with unrestricted access to rodent food and water. For parasite maintenance, each mouse was infected subcutaneously with 120 *S. mansoni* cercariae.

#### 2.9.2. In Vivo Antischistosomal Studies

For in vivo efficacy studies, female mice were each infected subcutaneously with 80 *S. mansoni* cercariae. Then, animals were randomly divided into experimental groups (five mice per group), and asiaticoside and PZQ were administered at single oral doses (400 mg/kg), by oral gavage, 49 days post infection (adult worm stage, patent infection) [1,2,3]. Group I: asiaticoside (400 mg/kg), which was dissolved in 2% ethanol in PBS (*v*/*v*); group II: control group, which was infected and received treatment only with vehicle (2% ethanol in PBS *v*/*v*); and group III: PZQ (400 mg/kg, p.o.) as a reference drug. The doses used of asiaticoside and PZQ were based on the protocols recommended for experimental schistosomiasis [1,2,3]. Two weeks after treatments (day 63 post infection), animals in all groups were euthanized using CO_2_.

To ensure that all parasites had been collected, schistosomes in the mesenteric veins were collected by portal perfusion, counted and sexed as previously described [30,31,32,33]. The assessment of the therapeutic efficacy was determined by comparing the worm reduction in the treated animals relative to the worm burden in the control group (treated with vehicle) [2,3], as well as based on the percentages of different egg developmental stages (oogram pattern), in which eggs, at different stages of maturity, were identified and the mean number of each stage was calculated [2,3].

#### 2.9.3. Randomization and Blinding

Animals were randomly assigned to their experimental groups and euthanized in a random manner with in vivo treatments counterbalanced randomly as well, in accordance with the standard operating procedures. All parameters were conducted by different investigators, conducted by two different people according to standard procedures [30,31,32,33] in compliance with the National Centre for the Replacement, Refinement, and Reduction of Animals in Research (NC3Rs) ARRIVE guidelines.

#### 2.9.4. Statistical Analysis

All statistical analyses were performed using Graph Pad Prism software 7.0. For in vivo experimental analysis, a parametric Dunnett’s multiple-comparison test was used to analyze the statistical significance of differences between mean experimental and control values. *p*-values of < 0.05 were considered significant. The data and statistical analysis are in accordance with the recommendations in the pharmacology field [30].

## 3. Results

### 3.1. Optimization of Incubation Conditions with Potato Apyrase

**Figure 1:**
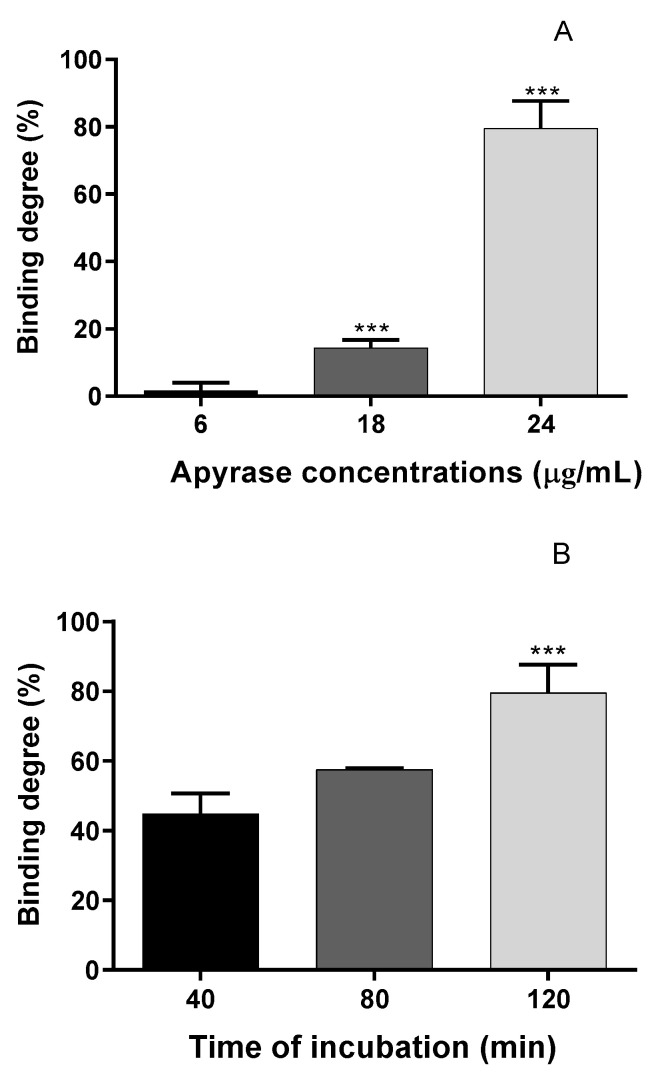
Effects of the incubation time and enzyme concentration on the binding ability between cardamonin and apyrase. (**A**) Binding degrees of cardamonin with apyrase at different concentrations. Cardamonin incubated with 6, 18, and 24 μg/mL of apyrase; *** *p* < 0.001 compared with group of 6 μg/mL of apyrase using the Dunnett’s multiple-comparison test. (**B**) Binding degrees of cardamonin with apyrase at different incubation times. *** *p* < 0.001 compared with group apyrase 40 min using the Dunnett’s multiple-comparison test.

Enzyme concentration and incubation time were optimized by studying the binding degree of cardamonin and apyrase. Firstly, different concentrations (6, 18 and 24 µg/mL) of apyrase were incubated with cardamonin (400 µM) for 120 min, at 37 °C. After ultrafiltration, the ultrafiltrates were analyzed by HPLC-DAD and the respective peak areas were used to calculate the binding degree. The cardamonin-apyrase binding percentages were 2%, 15%, and 79% after incubation with 6, 18 and 24 µg/mL of apyrase, respectively (Figure 1A). The higher binding degree was maximized by incubation of cardamonin with 24 µg/mL of apyrase, which concentration was used for subsequent investigation of the optimal incubation conditions. In addition, the incubation time was also evaluated. Then, cardamonin was incubated with 24 µg/mL of apyrase, at 37 °C, for 40, 80 and 120 min. The effect of incubation time on binding degree is shown in Figure 1B. Then, binding of cardamonin reached a maximum at 120 min, with 24 µg/mL of apyrase, which was the optimum incubation conditions to screen apyrase inhibitors in subsequent assays with *C. erecta* extract.

### 3.2. Identification of Potato Apyrase Ligands Using Ultrafiltration UHPLC-MS-QTOF Analysis

**Figure 2:**
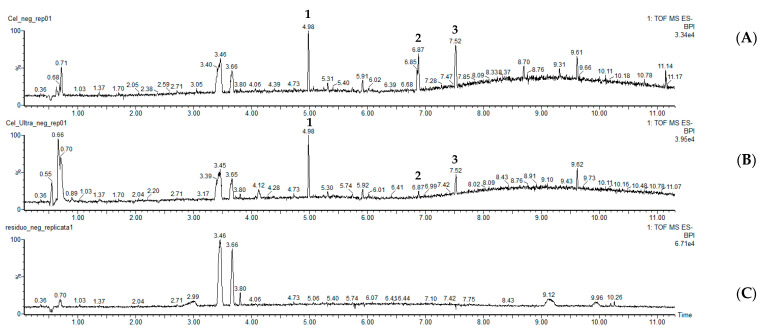
Representative UHPLC-ESI-QTOF-MS chromatograms of *C. erecta* extracts in the negative mode. (**A**) UHPLC-ESI-QTOF-MS chromatogram of the crude *C. erecta* extract without ultrafiltration; (**B**) representative UHPLC-ESI-QTOF-MS chromatogram of the ultrafiltrates of *C. erecta* extract after incubation with potato apyrase; (**C**) UHPLC-ESI-QTOF-MS chromatogram of the blank control group (ultrafiltrates of solvent with potato apyrase). The main screened potential apyrase ligands are marked with numbers above the peaks.

**Figure 3:**
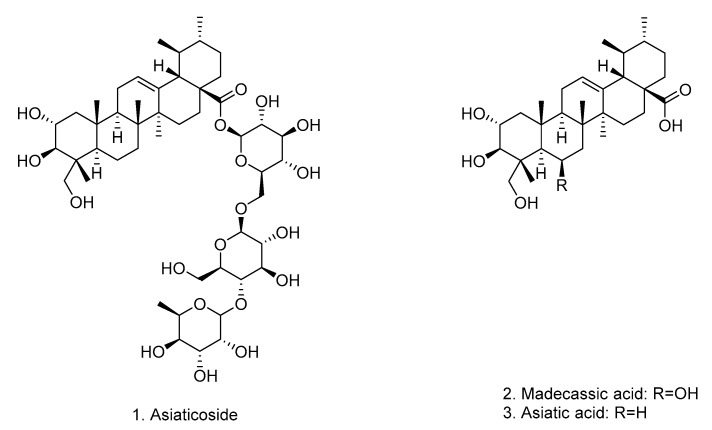
Chemical structures of compounds annotated in *C. erecta* by UHPLC-ESI-QTOF-MS analysis.

The *C. erecta* extract (Ce, 40 mg/mL) was submitted to incubation with apyrase, in the optimized incubation conditions, and subsequent UF-UHPLC-MS-QTOF analysis. The components of *C. erecta* ultrafiltrates were separated and analyzed by UHPLC-MS-QTOF. The ESI- mode was chosen based on previously LCMS studies with *Centella* extracts which showed that their compounds exhibit higher responses in negative ion mode [34,35]. Typical UHPLC-MS-QTOF chromatograms of *C. erecta* samples, at negative ion mode, are shown in Figure 2. Asiaticoside (**1**) was identified and other two components (Figure 3) were annotated by LC/MS analysis, as shown in Table 1. The data shown in Table 1 are consistent with the findings from other previous LC/MS studies with *Centella* species [36,37]. On mass spectrum, it was observed that asiaticoside (**1**, Rt = 4.98 min) predominantly formed chlorine [M + Cl]^−^ adduct ion in the negative mode with *m*/*z* 993.4827. The other fragment ions were at *m*/*z* 487.3400 [M-H-2Glc-Rha]^−^ and at *m*/*z* 469.1537 [M-H-2Glc-Rha-H_2_O]^−^, which are in accordance with MS/MS studies and fragmentation pattern previously reports to asiaticoside [36,37]. Similarly, peaks **2** and **3** were annotated as madecassic acid (Rt = 6.87 min) and asiatic acid (Rt = 7.52 min) (Figure 3), based on their negative molecular ions [M − H]^−^ at *m*/*z* 503.3387 [M − H]^−^ and at *m*/*z* 487.3445 [M − H]^−^, respectively, which agrees with the literature [36,37]. However, the structures of other possible ligands presented in the *C. erecta* extract, such as the compound at Rt = 9.61 min, were not successfully annotated and remain to be clarified.

### 3.3. Isolation of Asiaticoside from C. erecta Extract

As asiaticoside (Figure 3) was identified as one of the target ligand compounds, it was isolated from the *C. erecta* extract by chromatographic fractionation and additionally identified by ^1^H- and ^13^C-NMR data analysis in comparison to literature [38]: ^1^H NMR (500 MHz, CD_3_OD) δ (ppm): 5.31 (1H, d, J = 8.1 Hz, H-1′); 5.26 (1H, t, J = 3.4 Hz, H-12); 4.39 (1H, d, J = 7.9 Hz, H-1″); 3.57 (1H, d, J = 9 Hz, H-3); 2.25 (1H, d, J = 10.9 Hz, H-18); 1.30; 0.71; 1.07; 0.85; 1.14 (s, H-23, H-24, H-25, H-26 and H-27); 1.27 (3H, d, J = 6.2 Hz, H-6‴); 0.99 (3H, s, H-30); 0.92 (3H, d, J = 6.5 Hz, H-29).13C NMR (125 MHz, CD3OD) δ (ppm): 224.0 (C-11); 178.0 (C-28); 139.3 (C-13); 126.9 (C-12); 104.5 (C-1″); 102.9 (C-1‴); 96.8 (C-1′); 79.5 (C-3); 78.2 (C-5′); 77.9 (C-3′); 76.9 (C-3″); 76.7 (C-2″); 75.3 (C-5″); 73.8 (C-4‴); 73.7 (C-2′); 72.4 (C-4″); 72.2 (C-3‴); 71.0 (C-5‴); 70.6 (C-4′); 69.7 (C-23); 66.3 (C-2); 61.9 (C-6″); 59.7 (C-6′); 54.1 (C-18); 48.2 (C-9); 48.1 (C-1); 44.2 (C-4); 43.4 (C-14); 40.9 (C-8); 40.4 (C-19); 40.2 (C-20); 38.9 (C-10); 37.6 (C-22); 33.6 (C-7); 31.7 (C-21); 29.3 (C-15); 25.2 (C-16); 24.5 (C-27); 21.6 (C-30); 19.1 (C-6); 18.8 (C-6‴); 17.9 (C-29); 17.8 (C-26); 17.6 (C-25); 13.9 (C-24). HRMS (ESI) *m*/*z*: 993.4827 [M + Cl]^−^ found; 993.4831 [M + Cl]^−^ calculated. Purity of the isolated asiaticoside was estimated to be higher than 95% by ^1^H NMR data analysis.

### 3.4. Determination of Apyrase Activity of Asiaticoside

**Figure 4:**
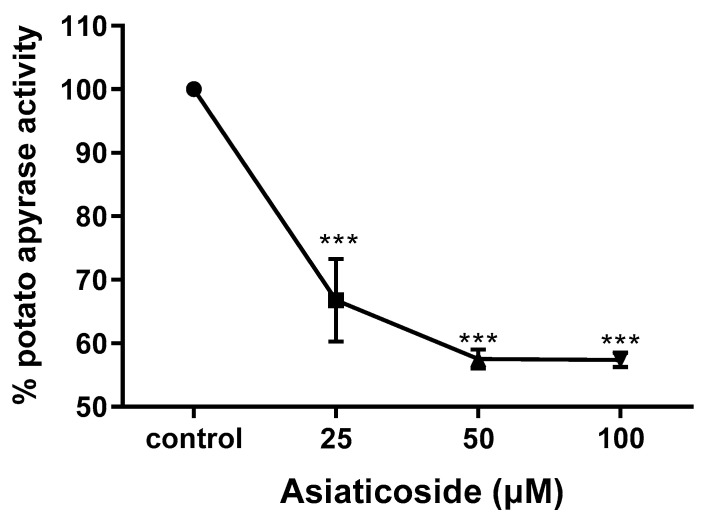
Effects of asiaticoside on the ATPase activity of potato apyrase. Asiaticoside was assessed based on the activity observed for the control, which represents 100% of the ATPase activity. For each drug concentration, a DMSO control was performed. Basal activity in the absence of asiaticoside or DMSO was 22.053 μmol Pi·mg^−1^·min^−1^. Each analysis represents an experiment done in triplicate. *** *p* < 0.001 compared with control group using the Dunnett’s multiple-comparison test.

After isolation, the inhibitory activity of asiaticoside (25, 50 and 100 µM) against potato apyrase was investigated. Asiaticoside was able to decrease the enzymatic activity of potato apyrase (Figure 4). Although it was not possible to calculate the IC_50_ value, asiaticoside inhibited potato apyrase activity by approximately 43% at 100 µM, while at 50 µM potato apyrase activity was inhibited by about 33% (Figure 4).

### 3.5. Molecular Docking Analysis of Asiaticoside against Potato Apyrase, SmNTPDase 1 and SmNTPDase 2

**Figure 5:**
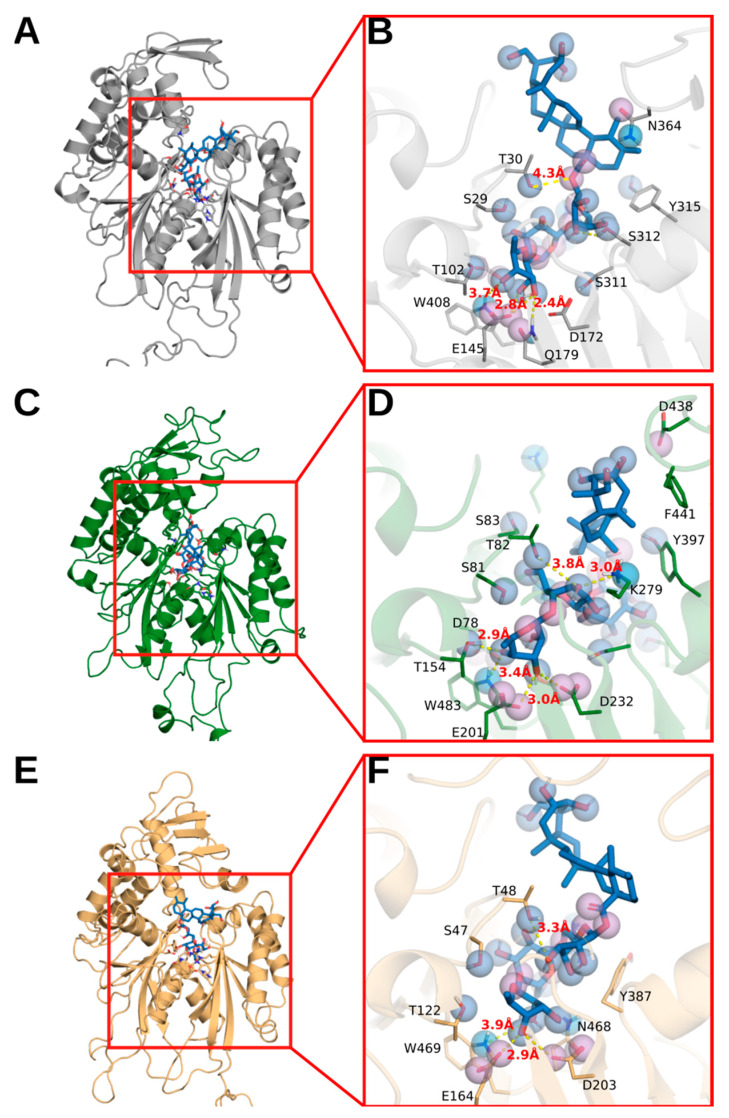
Molecular docking assays of asiaticoside and NTPDases. In (**A**,**C**,**E**) are represented the ECD domain of apyrase, SmNTPDase 1, and SmNTPDase 2, respectively. (**B**,**D**,**F**) show the best conformations of asiaticoside, using Autodock Vina, for apyrase, SmNTPDase 1, and SmNTPDase 2, respectively. Spheres represent pharmacophoric characteristics: hydrogen bond donor (light blue), hydrogen bond acceptor (purple), hydrogen bond donor and acceptor (blue).

To investigate possible modes of asiaticoside interaction with target enzymes at the active sites, we performed molecular docking simulations. As shown in Table 2, asiaticoside binds to the active sites of all evaluated NTPDases enzymes, SmATPDase1, SmATPDase2 and apyrase, with negative energy (affinity) values, suggesting that these bindings occur spontaneously. Furthermore, asiaticoside can perform hydrogen bonding interactions with donor and acceptor residues at the nucleotide-binding site of all target enzymes. Figure 5 shows the hydrogen bonding or strong electrostatic interactions (2.2 Å–3.2 Å) that asiaticoside performs with the catalytic residue of glutamic acid (E145, E201, and E164 from apyrase, SmNTPDase1, and SmNTPDase2, respectively), which activates the nucleophilic water and performs the hydrolysis in the terminal phosphate of the substrate [39]. Asiaticoside also performs weak electrostatic interactions (3.3 Å–4.0 Å) with serine or threonine residues (T30, S81, and T48 from apyrase, SmNTPDase1, and SmNTPDase2, respectively) which carry out the hydrolysis of the β phosphates of the di- and triphosphate nucleotides [40]. In addition, a conserved tryptophan residue, located at the catalytic site, could play an important role as hydrogen donor to asiaticoside.

### 3.6. In Vivo Antischistosomal Studies of Asiaticoside against S. mansoni in Patent Infection

**Figure 6:**
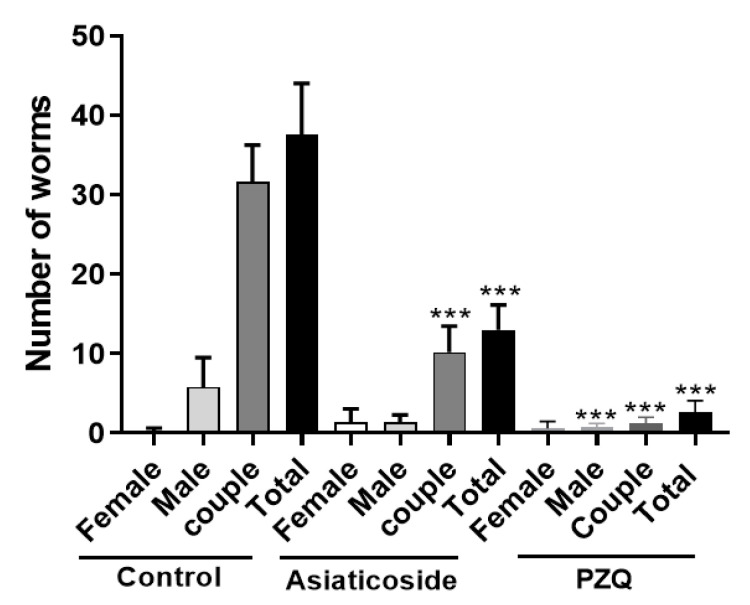
Effects on worm burden of single doses of asiaticoside (400 mg/kg, p.o) and PZQ (400 mg/kg, p.o) administered to mice harboring a 49-day-old adult *S. mansoni* infection, stratified by sex. Bars (mean ± SD) represent data from five individual mice that were infected and treated with asiaticoside, PZQ or vehicle (control) *** *p* < 0.001 compared with group treated with vehicle (control) using the Dunnett’s multiple-comparison test.

**Figure 7:**
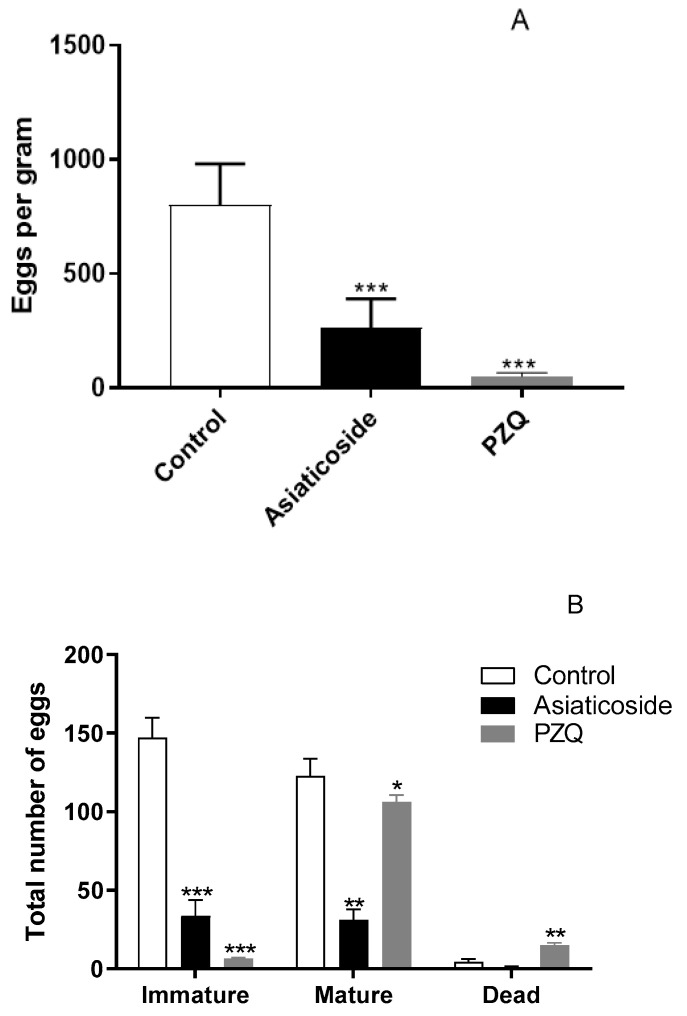
Effects on stool egg load (**A**) and on egg development stages (oogram) (**B**) of single doses of asiaticoside (400 mg/kg, p.o.) or PZQ (400 mg/kg, p.o.) administered to mice harboring a 49-day-old adult *S. mansoni* infection. Bars (mean ± SD) represent data from five individual mice that were infected and treated with asiaticoside, PZQ or vehicle (control). *** *p* < 0.001, ** *p* < 0.01, and * *p* < 0.05, compared with group treated with vehicle (control), using the Dunnett’s multiple-comparison test.

Asiaticoside was in vivo evaluated in a murine model of schistosomiasis. After oral treatment, asiaticoside (400 mg/kg) showed a significant total worm burden reduction of 65.4% (*p* < 0.001) in comparison with control group (Figure 6). PZQ (400 mg/kg, p.o.), used as a reference drug, resulted in total worm burden reduction of 93.1% (*p* < 0.001) (Figure 6). In addition, the couple worms load was reduced by 67.7% (*p* < 0.001) after treatment with a single oral dose of asiaticoside (400 mg/kg) in comparison with control group (Figure 6). In feces collected from infected treated mice, the number of eggs per gram was reduced by 67.7% (*p* < 0.001) and 93.6% (*p* < 0.001) after the oral treatment with asiaticoside and PZQ, respectively (Figure 7A). Additionally, in the analysis of egg development stages (oogram), the number of immature eggs was reduced in 71.5% (*p* < 0.001) and 94.4% (*p* < 0.001) after the oral treatment with asiaticoside and PZQ, respectively (Figure 7B).

## 4. Discussion

The discovery of new antischistosomal compounds is crucial in terms of public health, since schistosomiasis remains as the second most important human parasitic disease [1,4,41]. Due to the imperative need to identify new drugs, several natural compounds have been recently studied against *S. mansoni* [1,6]. Additionally, our previous studies have shown that cardamonin has in vitro and in vivo antischistosomal activity against *S. mansoni*, also being effective in inhibiting both potato apyrase and SmNTPDases [13]. In addition, in a preliminary assessment with some plant extracts by our group, the hydroalcoholic extract of *C. erecta* showed as a potential source of apyrase inhibitors (data not shown). Based on these findings and corroborating with the high homology degree between SmNTPDases and potato apyrase [13], we used a UF-UHPLC-QTOF method to identify potential apyrase inhibitors from the *C. erecta* extract.

Typically, an affinity ultrafiltration LC-MS screening involves three steps: incubation, screening by ultrafiltration and characterization by MS [42]. It is known that the optimization of conditions may increase the accuracy of the UF-UHPLC-ESI-QTOF-MS results [18]. Then, first, different incubation conditions were optimized by comparing the binding degree of cardamonin with potato apyrase to achieve favorable screening conditions to maximize the isolation of potential ligands. After, screening assays by UF-UHPLC-QTOF-MS with *C. erecta* extract were carried out for identification of apyrase ligands. The particular ligands from *C. erecta* extract may bound to the apyrase sites, forming enzyme-ligand complexes during the incubation process, which, due to their macromolecular size, would remain in the ultrafiltration membrane when centrifuged, while the unbound small compounds could pass through the membrane [43]. After, the ultrafiltrates of the *C. erecta* were subjected to UHPLC-ESI-QTOF-MS identification. According to UHPLC-ESI-QTOF-MS analysis, the ultrafiltration process produced an ultrafiltrate enriched mainly with three compounds (peaks 1–3) with binding affinity for apyrase, although a little difference between the LCMS chromatograms of the crude *C. erecta* extract and the ultrafiltrate was observed. These main compounds in ultrafiltrates were analyzed by their mass spectrometry data, accurate molecular weight, and fragment ions and annotated as asiaticoside (peak **1**), madecassic acid (peak **2**), and asiatic acid (peak **3**). Although other possible ligands might be presented in the *C. erecta* extract (such as in Rt= 9.61 min), only three target compounds were annotated as potential apyrase inhibitors. Among them, asiaticoside was the only successfully isolated from *C. erecta* extract by chromatographic fractionation and further chemically identified by ^13^C- and ^1^H- NMR data analysis. *Centella* species consist mainly of triterpenoid saponins, in which asiaticoside is one of most studied compounds [36].

However, binding of a ligand target compound to the enzyme does not mean that it is an enzyme inhibitor, due to the possibility of non-specific binding of the compound to non-functional sites on the enzyme [19]. Then, in alignment with the previously outlined screening strategy, the apyrase inhibitory activity of asiaticoside was in vitro evaluated, showing a moderate apyrase inhibition for asiaticoside.

Additionally, to investigate how asiaticoside might bind to the active sites of NTPDases enzymes, asiaticoside was docked to active pockets of potato apyrase and SmNTPDases 1 and 2. Our results indicate that asiaticoside is able to interact with important catalytic residues of apyrase and SmNTPDases, providing a possible inhibition of these target proteins. In addition, we also observed that asiaticoside preferably interacted in the positive electrostatic region of the catalytic site from target proteins, similar that results obtained to cardamonin [13].

Then, considering the importance of apyrase inhibitors as potential antischistosomal compounds and keeping in view the above-mentioned experimental approach, we evaluate the in vivo activity of asiaticoside in the *S. mansoni* mouse model. The in vivo treatment with asiaticoside was performed in mice harboring patent *S. mansoni* infection. To our knowledge, asiaticoside has not been yet evaluated against *Schistosoma* sp. After oral treatment at a single dose (400 mg/kg) of asiaticoside, worm burden was significantly reduced in comparison with the control group. Additionally, when compared to the reference drug PZQ (400 mg/kg), which reduced the parasitic burden in ~90% [30], asiaticoside exhibited a meaningful antischistosomal efficacy. Regarding the toxicity profile, previous studies have shown that both asiaticoside and standardized *Centella* extracts (with high concentration of asiaticoside) did not show any adverse effects by oral administration in mice at least up to 1 g/kg [44,45]. Furthermore, a standardized *C. asiatica* extract (containing ~46% of asiaticoside) exhibited LD_50_ > 2 g/kg by oral administration in rats [46]. In addition, it was showed that unlike asiatic acid (aglycone of asiaticoside), asiaticoside exhibits no hemolytic effects [47].

Moreover, there are few reports in the literature regarding the schistosomicidal activity of saponins. Previous studies showed that saponins from the marine organisms *Actinopyga echinites* and *Holothuria polii* have interesting in vitro schistosomicidal activity [48]. Also, in a zoopharmacological study in chimpanzees, steroidal saponins from *Vernonia amygdalina* (Asteraceae) were in vitro and in vivo evaluated against *S. japonicum* [49], showing moderate activity. In addition, the natural saponin hederacoside, isolated from the *Pulsatilla chinensis* (Bunge) Regel, exhibits antischistosomal properties in vivo against *S. japonicum* and *S. mansoni* [50].

In addition, it is known that the main lesions caused by *S. mansoni* to the organism are due to eggs in the host’s tissues, triggering inflammatory reactions and granuloma formation [51,52]. Then, considering that eggs are directly associated with the transmission and the immunopathogenesis of schistosomiasis [51], the therapeutic efficacy was also assessed by oogram and egg load in mice harboring adult *S. mansoni*. After the treatment with asiaticoside the number of eggs per gram (OPG) was also markedly reduced in feces examined by the Kato–Katz method. Moreover, the oogram pattern showed that the oral administration of asiaticoside produced significant reductions in immature and mature eggs. Taken together, our results on egg burden agree with the noticeable ability of asiaticoside in reducing adult worms of *Schistosoma*.

The *Schistosoma* tegument is the interface between the environment and the parasite, achieving vital functions for the worms, such as nutrient absorption, excretion, protection against the host’s immune system [31]. In *Schistosoma* tegument are some essential enzymes, such as the SmNTPDases, which are important for the survival of *Schistosoma* worms inside the host [12,31,53]. Then, considering the mechanism of action, although a possible inhibition of SmATPDases by asiaticoside might be present, it cannot discard other effects. Since asiaticoside may exert multiple biological properties [54], other mechanisms might contribute to its antischistosomal action. In this regard, it is also reported that after oral administration of asiaticoside, it may undergo biotransformation into asiatic acid, the aglycone of asiaticoside that was also annotated as apyrase ligand in our ultrafiltration screening. Then, the hypothesis that asiatic acid may also have antischistosomal activity [55] should be further explored in the future. Thus, the precise in vivo antischistosomal mechanisms of asiaticoside must be further investigated. Furthermore, our present data are consistent with and encourage further exploration of apyrase inhibitors to search for new antischistosomal agents.

## 5. Conclusions

In this study, an ultrafiltration combined with UHPLC-QTOF-MS approach was used to identify the asiaticoside as a potential apyrase inhibitor from *C. erecta* extract. Madecassic and asiatic acids were also annotated as apyrase-binding compounds by this method. Asiaticoside was isolated from *C. erecta* extract and further in vitro assays confirmed its potato apyrase inhibitory activity. Additionally, molecular modeling and docking assays were used to evaluate the interaction of asiaticoside with the active sites of SmNTPDase 1, SmNTPDase 2, and potato apyrase. In addition, our findings demonstrated, for the first time, that asiaticoside has markedly in vivo antischistosomal efficacy in mice harboring a patent *S. mansoni* infection, markedly decreasing the total worm load and egg burden. However, more clinical studies are needed to validate the use of asiaticoside as a therapeutic schistosomicidal agent. Additionally, future studies are also in progress to evaluate the antischistosomal properties of madecassic and asiatic acids.

## Figures and Tables

**Table 1:** Chemical characterization of *C. erecta* ultrafiltrates by UHPLC-ESI-QTOF-MS.

Peak	Proposed Compounds	Rt (min)	*m*/*z* Experimental [M − H]^−^	Main Fragments via MS/MS	Molecular Formula	References
1	Asiaticoside	4.98	993.4827	957.5045; 487.3400; 469.1537; 162.8393; 160.8240; 116.9285	C_48_H_78_O_19_	[36,37]
2	Madecassic acid	6.87	503.3387	325.1851; 178.8417; 162.8393; 160.8420; 116.9285	C_30_H_48_O_6_	[36,37]
3	Asiatic acid	7.52	487.3445	441.2468; 325.1851; 178.8417; 162.8419; 160.8420; 116.9285	C_30_H_48_O_5_	[36,37]

**Table 2:** Molecular docking results for NTPDases target proteins. The energy and cKi values as well as interactions represent the asiaticoside pose with best protein contacts.

Protein	Energy ^a^	*cKi* ^b^	Interactions
Apyrase	−9.9	0.055	T30, E145, D172, S312, Q179, W408
SmNTPDase 1	−10.0	0.047	S81, T154, E201, D232, K279, W483
SmNTPDase 2	−11.3	0.005	T48, R51, H53, H76, E164, D203, E466, N468, W469

^a^ Best energy binding mode in kcal/mol. ^b^ Theoretical *Ki* in μM (*cK_i_*) calculated according to literature [28].

## Data Availability

Data is contained within the article.
